# Health Development versus Medical Relief: The Illusion of Aid

**DOI:** 10.1371/journal.pmed.0030507

**Published:** 2006-11-28

**Authors:** Maria Rubio, Jose Luis Portero

**Affiliations:** Madrid, Spain

Gorik Ooms sheds light on the controversial use of the concept of sustainability by development agencies [1]. International organizations involved in foreign aid and local governments support health policies that decrease the size, the potential, and the budget of the public health sector and hamper the access to health through fee-for-service systems. Under the so-called Health Sector Reform Agenda, out-of-pocket charges make unsustainable the cost of health care for the poorest. Health insurance systems have a minor effect on the health of the poor, either because the system generates permanent barriers to access health care or the services are not really functioning. It is debatable whether a complex instrument, such as health insurance, can be valuable in societies with depleted governmental funds, rundown health services, and a high index of poverty and inequity. So, free health care is the only affordable and sustainable option for the poorest.

On the other hand, Médecins Sans Frontières (MSF) fosters the illusion of independent aid in a vast world full of necessities, makes irrelevant political views through the flag of neutrality, and digests the lecture of conflicts for the taste of the white man's tears. MSF knows that their projects, as most of the projects, are not sustainable and therefore rejects sustainability as an argument to act. But MSF is concerned about sustainability as the organization calls for stronger national commitment and more international aid for health through emergency development. Behind the oratory, MSF assumes that the governments have the power for an appropriate answer.

Sustainability cannot be analyzed alone. Community development, grassroots democracy, social justice, human rights, humanitarianism, or empowerment are the conceptual ideas that back the illusion of aid in the Western civil society. They are rhetorical terms that could even sound radical but, paradoxically, due to their apolitical approaches, make international aid and cooperation harmless for governments and policies that generate injustice. Moreover, postmodernism appeals for individual attention and marginalizes collective political actions that could have greater impact.

Solomon R. Benatar wrote in this journal that poor health reflects systemic dysfunction in a complex world and calls the attention to address the complex system forces that sustain poverty and poor health [2]. We are far from addressing these complex system forces that themselves remain, unfortunately, in good health. At the end, we might need politics rather than the illusion of independent aid.
